# Clinical Validation of M-TONX: A Novel Combo Rebound Tonometer and Pachymeter

**DOI:** 10.1167/tvst.13.12.34

**Published:** 2024-12-26

**Authors:** Naveed Nilforushan, Mozafar Yousefi, Ankica Babic, Arash Gharehbaghi

**Affiliations:** 1 Eye Research Centre, Iran University of Medical Sciences, Tehran, Iran; 2 Moj Teb Parsian Incorporation, Tehran, Iran; 3 Department of Biomedical Engineering, Linköping University, Linköping, Sweden; 4 Department of Information Sciences and Media Studies, University of Bergen, Bergen, Norway

**Keywords:** intraocular pressure (IOP), central corneal thickness (CCT), tonometry, pachymeter, rebound

## Abstract

**Purpose:**

This study aims to perform a clinical investigation of an innovative rebound technology-based device, the M-TONX, to simultaneously measure intraocular pressure (IOP) and central corneal thickness (CCT).

**Methods:**

The IOP and CCT of the patients were first measured by the M-TONX. Then, the measurements were repeated by the Goldman applanation (GAT) and the Pentacam corneal topographer, as the standard devices. For the statistical analysis, the patients were stratified based on their IOPs to group 1 (IOP < = 16 millimeters of mercury [mm Hg]), group 2 (16 mm Hg < IOP < 23 mm Hg), and group 3 (IOP > = 23 mm Hg). The stratification was also performed for the CCTs as: class 1 (CCT < = 475 µm), class 2 (475 < CCT < 574 µm), and class 3 (CCT > = 575 µm).

**Results:**

Of the 374 eyes (225 subjects and 43% women), 262, 66, and 46 eyes belonged to group 1, group 2, and group 3, respectively. Very high IOP (>35 mm Hg) was observed in 12 eyes. The overall confidence interval of the deviation (confidence level = 95%) from the standard devices was estimated to be 0.7 to 1.2 mm Hg for IOP, and −12.7 to −5.4 µm for CCT. The stratified analysis showed substantial agreement with the standard devices with the intraclass correlation > 0.65 and the Pearson Correlation > 0.8 calculated for all the groups and classes.

**Conclusions:**

The M-TONX exhibited a reliable performance concerning the standards for measuring IOP and CCT. Its accuracy remains stable for a broad range of IOP and CCT. The M-TONX successfully incorporates two separate functionalities into a single compact user-friendly device.

**Translational Relevance:**

This study uncovers the conformity of the technology with the standards, linking fundamental research to clinical care.

## Introduction

Intraocular pressure (IOP) and central corneal thickness (CCT) are the two fundamental parameters commonly used by ophthalmologists in many of the clinical investigations.^1^^,^^2^ Accurate measurement of these two essential parameters plays an important role in the management of patients with or at risk of glaucoma.^3^ Accurate measurement of IOP in a patient-friendly manner has been a challenge for ophthalmologists, particularly with measurements for patients with thick or thin cornea or with a history of corneal refractive surgeries.^4^^–^^11^ Rebound technology, under the brand name of iCare, has been introduced as a valuable and efficient method for measuring IOP.^12^^–^^15^ However, the accuracy of this device is controversial due to its limitations in accounting for CCT and the biomechanical properties of the cornea, particularly in patients with significant deviations from the normal CCT range. The M-TONX is a combination rebound tonometer and pachymeter that has recently been introduced in clinical settings as a compact device for measuring IOP, CCT, and estimating the true-IOP based on the measured CCT and biomechanical characteristics of the cornea. Here, we present the results of a clinical validation trial on the M-TONX, conducted in accordance with ISO 8612, as the specific standard for tonometer. The inclusion and exclusion criteria for the patient group were designed closely following this standard.

## M-TONX Device

**Figure 1.**
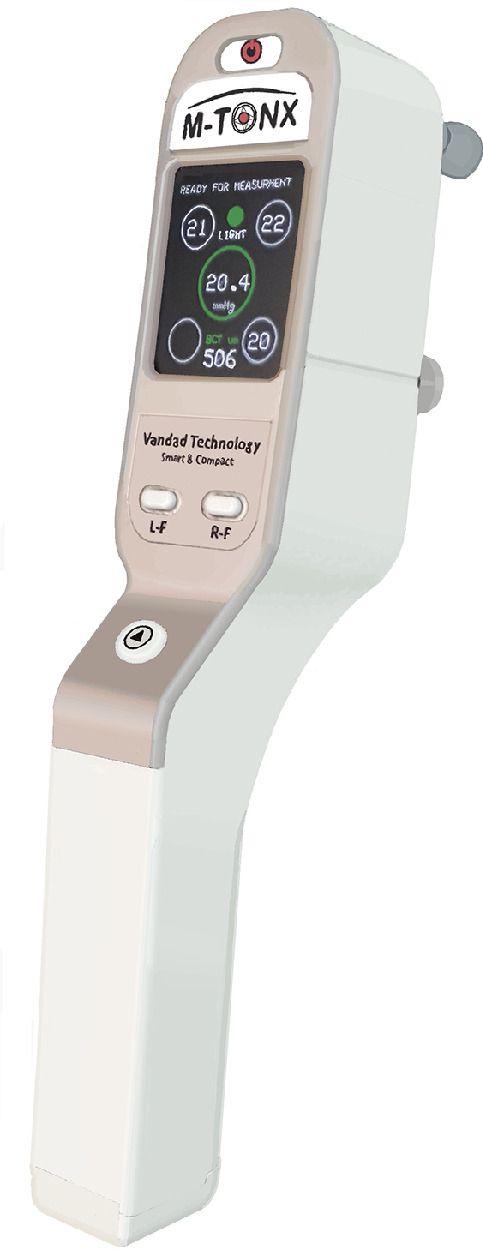
The M-TONX device.

The M-TONX is a newly introduced device for simultaneous measurement of IOP and CCT. The measurement technology is composed of a bound and rebound sensor setup, along with the sophisticated signal processing and machine learning methods used to estimate IOP and CCT. Figure 1 depicts the M-TONX device (Moj Teb Parsian Incorporation, Tehran, Iran), which incorporates the rebound sensor setup.

The technology used for the development of the M-TONX has been internationally patented (US 11,925,413 B2). In this technology, the sensor throws a tiny probe with a round tip of 1.8 mm. The probe touches the center of the cornea for a maximum of 8 milliseconds (m/s) with a maximal speed of 0.4 m/s. The ferromagnetic property of the metal trail of the probe provides acquisition of subtle details of corneal characteristics, such as corneal biomechanics, using a patient-specific model uniquely obtained for each patient. To adjust the device on the center of the cornea during measurement, a light source is positioned and centered with the path of the probe. This improves the accuracy of the measurements and provides greater stability on the measuring spot. The device performs four independent measurements to the operators, and the estimated value of IOP and CCT are calculated based on the average value of the measurements. The variance of the measurements is used as an indicator of measurement quality, with low-quality measurements highlighted in red color.

A set of the statistical pattern recognition methods processes the signal acquired from the sensor to estimate the IOP and CCT values. The M-TONX uses a combination of the unsupervised classification method and recurrent neural network for preprocessing and segmenting the sensor signal after being digitally filtered. The biomechanical parameters as well as IOP and CCT values are obtained by applying the nonlinear time invariant regression method to the segmented time series. This enables the device to demonstrate the true-IOP value, by compensating the effect of the corneal thickness in the IOP measurement. The M-TONX displays IOP, CCT, and true-IOP, automatically after the fourth measurement. The IOP value is directly measured by the device, whereas the true-IOP is derived from a model that compensates for corneal characteristics. Although the M-TONX displays the true-IOP, ophthalmologists continue to use the conventional IOP value, as it aligns better with reference devices currently used in clinical settings, such as the Goldmann applanation tonometer (GAT).

## Methods

### Study Compliance

This study was conducted in accordance with the international standard UNI EN ISO 14155:2011, which outlines good clinical practice for clinical investigations of medical devices involving human subjects. This facilitated the exchange of performance study results among countries in the European Union and those outside the Union. The study adhered to the most recent version of the World Medical Association Declaration of Helsinki on Ethical Principles for Medical Research Involving Human Subjects, which helped ensure compliance with the regulatory requirements.^16^

### Experimental Design

The clinical investigation was conducted at the ophthalmology center of Iran University of Medical Sciences, Rasool e Akram Hospital, Tehran, Iran. The study protocol was reviewed and approved by the review board and the ethics committee of Iran University of Medical Sciences prior to the data collection (IR.IUMS.REC.1400.1193). The informed consent was obtained from the participants prior to the recruitment. In this investigation, the IOP and the CCT were measured once by the M-TONX. The GAT and Pentacam topography served as the reference devices for measuring the IOP and the CCT-keratometry, respectively, at the hospital. This study focused solely on the standard IOP and CCT values. Hence, the true-IOP value reported by the M-TONX was not taken into account in the investigation. It is clear that the GAT cannot serve as an adequate reference device for true-IOP.

### Study Subjects

This study was designed as an all-comers study except for the patients who met one or more of the following criteria:

• Irregular corneal astigmatism, which affects the precision of applanation tonometry either as a primary condition or secondary to trauma, infection or surgery.
• Ocular surface infection and inflammation.
• Corneal scar in the central 5 mm of cornea.
• Those who were not willing to sign the informed consent.

### Measured Parameters

The IOP was measured twice in both eyes; first with the M-TONX and then with the GAT, within a 5-minute interval. If the difference between the 2 measurements of each device was more than 2 millimeters of mercury (mm Hg), a third measurement was taken, and the average of the 3 measurements was recorded. CCT, keratometry, and the axis of astigmatism were also obtained from the Pentacam. Additional data such as date and type of previous ocular surgeries, ocular and systemic diseases, best corrected visual acuity, and topical or systemic medications were collected.

### Patient Stratification

The subjects were stratified into three groups based on their IOP according to the GAT measurements: group 1 (IOP < = 16 mm Hg), group 2 (16 mm Hg < IOP < 23 mm Hg), and group 3 (IOP > = 23 mm Hg). Patient recruitment continued until the following criteria were met:

• The total number of subjects of less than 500.
• At least 40 subjects of each group.
• At least 5 subjects of group 3, had IOP > 35 mm Hg according to the reference device.

This stratification was conducted in accordance with the directives of ISO 8612, which is considered as the major regulation for tonometer devices.

### Study Procedure

**Figure 2.**
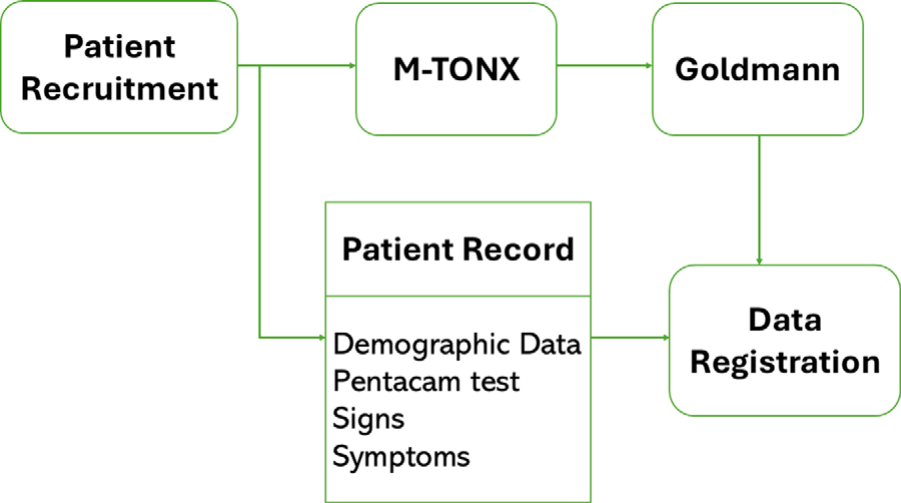
The workflow of the clinical procedure.

All patients who were referred to the glaucoma clinic between March 2022 and October 2022 were considered to participate in the clinical study. The study protocol is illustrated in Figure 2.

The recruited patients underwent the Pentacam imaging, followed by a combined IOP-CCT measurement using the M-TONX device. It is important to note that the Pentacam is recognized as a reliable device for CCT measurement with marginal deviations from the ultrasound pachymetry.^17^^,^^18^ Next, the IOP was measured using the GAT by another operator. The M-TONX and GAT operators were independent, and the GAT measurements were masked to the M-TONX operator. Unlike the applanation technology, which maintains continuous contact with the corneal surface, the rebound technology uses a spontaneous touch (a few milliseconds) to the corneal surface. This can affect the measurement accuracy in the presence of the persistent eye drops, such as anesthetic eye drops, that is needed for using the GAT. This was the primary motivation of using the M-TONX prior to the GAT in this study.

### Statistical Analyses

Data analysis and representation were conducted using the Python programming language within Jupiter Notebook platform. The software packages of “Statistics,” “Sklearn,” and “Scipy.stats,” were used for the statistical analyses. Regression analysis was conducted to explore the relationship between the values measured by the M-TONX and those obtained from standard devices. The agreement with the standard devices was quantitatively explored by different metrics: Pearson correlation, Spearman correlations, and Cohen_Kappa. Unlike the Pearson correlation, the Spearman correlation uses the rank sum of the measurements to cope with the non-Gaussian distribution of the measurements. The Cohen_Kappa was calculated for the study groups to determine the intraclass correlation. Moreover, the reliability of the M-TONX measurements was quantified using the following formula:

$$\begin{array}{c} R=\frac{True\;Variance}{True\;Variance+Error\;Variance} \end{array}$$

where “True Variance” and “Error Variance” denotes the measurement variance of the reference device (in this study the standard devices: the GAT or the Pentacam) and deviation of the measurements from the standard devices, respectively. This metric has been utilized for various clinical validations.^19^ The Bland-Altman plot was invoked to visualize deviation of the M-TONX outcomes from the standard devices. Descriptive statistics of the deviations were estimated for the IOP and CCT, both for the entire study population, and for the stratified population independently. The normality of the statistical distributions was examined using Shapiro-Wilk test. The statistical *t*-test method was used for estimating the confidence interval of the deviation with the *P* value threshold of (<0.05%) as the accepted margin for the estimations. Sample size was calculated based on an error margin of ±1 mm Hg for the IOP, and 25 µm for CCT measurements with 95% of confidence interval.

## Results

A total of 225 individuals were enrolled by the study, with 374 eyes undergoing pairwise measurements using the M-TONX (with the red-colored results omitted) and the GAT. Out of the 374 eyes with the measures of IOP, a total of 226 eyes completed the entire examinations with the M-TONX, the GAT, and the Pentacam. The standard IOP measurements by the M-TONX, were explored in terms of the deviation from the GAT. The true-IOP displayed by the M-TONX was completely disregarded and did not influence the study. The patients were stratified into 3 groups according to their IOP values: group 1, group 2, and group 3, with IOP ≤ 16, 16 < IOP < 23, and IOP ≥ 23, respectively. The subjects covered a broad age range spanning from 5 to 86 years. These ranges were 14 to 86 years, 5 to 86 years, and 10 to 80 years for the group 1, group 2, and group 3, respectively. The subjects were further categorized into 3 classes based on their CCT values measured by the Pentacam: class 1, class 2, and class 3 with CCT ≤ 475 µm, 475 µm < CCT < 575 µm, and CCT ≥ 575, respectively. The age ranges of the referrals from the class 1, class 2, and class 3 were 30 to 81 years, 5 to 86 years, and 10 to 82 years, respectively. Table 1 represents the demography of the patient population.

**Table 1.** Demography of the Study Population in Terms of the Intraocular Pressure (IOP) and the Central Corneal Thickness (CCT)

Case	Number	Age, Range, Y	Age, Mean ± STD, Y
Total subjects	225	5–86	54.8 ± 20.1
Female subjects	97	5–85	53.4 ± 20.7
Male subjects	128	10–86	55.2 ± 20.3
Eyes with the completed IOP measurement	374	5–86	54.7 ± 19.7
Eyes with the completed CCT measurement	226	5–86	51.7 ± 20.3
IOP < = 16 mm Hg	262	14–86	57.0 ± 17.2
16 < IOP < 23 mm Hg	66	5–86	54.7 ± 19.7
IOP > = 23 mm Hg	46	10–80	45.4 ± 24.7
IOP > = 35 mm Hg	12	10–80	54.3 ± 20.1
CCT < = 475 µm	25	30–81	58.8 ± 19.8
475 < CCT < 575 µm	152	5–86	51.7 ± 20.3
CCT > = 575 µm	49	10–82	43.1 ± 22.5

**Figure 3.**
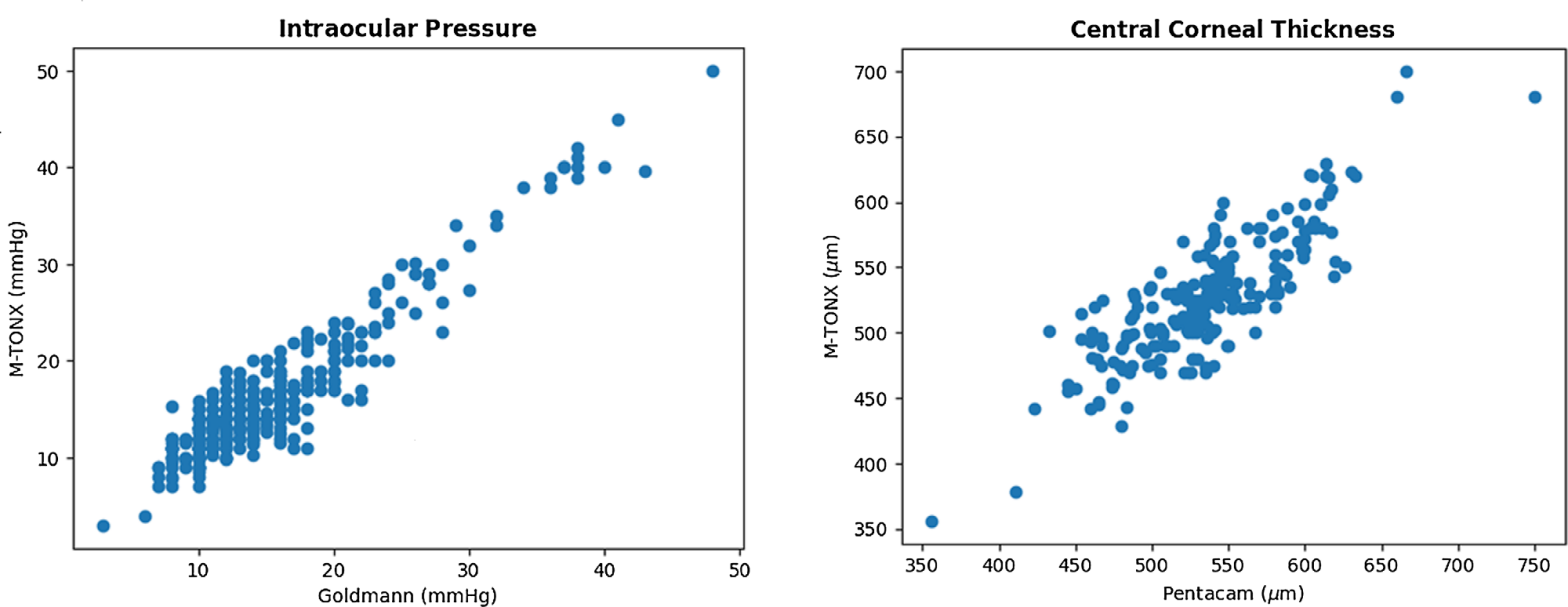
Regression plot of the M-TONX with the Goldmann applanation tonometer and the Pentacam for IOP (*left*) and CCT (*right*). Very strong correlations of *r* = 0.83 and *r* = 0.95 was observed for CCT and IOP, respectively.

The subjects were dominantly cases of glaucoma or glaucoma-suspect according to the clinical assessments. Deviation of the M-TONX from the reference devices was calculated for IOP and CCT. The scatter plot of IOP (the M-TONX versus the GAT), and the one of CCT (the M-TONX versus the Pentacam) are demonstrated in Figure 3.

The regression analysis exhibited very strong positive correlation between the M-TONX and the GAT measurements, with the correlation coefficient of *r* = 0.94 (*P* < 0.000). The correlation between the M-TONX and the Pentacam degraded, but still were considered as a very strong positive, with *r* = 0.83 (*P* < 0.000). It is worth noting that the grading standards define very strong correlation when the correlation coefficient exceeds *r* ≥ 0.8. The regression formulas for IOP and CCT were found to be as follows:

(1) $$\begin{array}{c} IOP_{GAT}=0.91*IOP_{MTONX}+0.43\; \end{array}$$

(2) $$\begin{array}{c} CCT_{Pentacam}=0.89*CCT_{MTONX}+69.38\; \end{array}$$

The agreement of the M-TONX measurements with the reference devices was explored using different metrics and represented in Table 2.

**Table 2.** Agreement Between the M-TONX Measurements and the Standard Devices, the Goldmann and the Pentacam, for the 3 Groups of Intraocular Pressure, Group 1 (IOP < = 16 mm Hg), Group 2 (16 < IOP < 23 mm Hg), and Group 3 (IOP > = 23 mm Hg), Together With the 3 Classes of Corneal Thickness: Class 1 (CCT < = 475 µm), Class 2 (457 < CCT < 575 µm), and Class 3 (CCT > 575 µm)

	IOP, mm Hg				CCT, µm			
	All	Group 1	Group 2	Group 3	All	Class 1	Class 2	Class 3
r	0.94	0.91	0.92	0.93	0.83	0.85	0.80	0.83
rs	0.84	0.78	0.81	0.83	0.80	0.82	0.75	0.80
Rl	0.89	0.87	0.87	0.82	0.76	0.78	0.76	0.77

The agreement is explored by three metrics: the Pearson correlation (r), the Spearman correlation (rs), and the Reliability (Rl).

A very strong agreement (>0.8) was found between the measured IOPs for all the groups. A considerable difference between Pearson and Spearman correlations was found for all the IOP groups, implying on the non-Gaussian statistical distribution of the data. Reliability was consistently very strong (>0.8) for all the three groups. Slight deviations were observed over the groups, showing a stable performance of the M-ONX tonometer over the three groups. Nevertheless, the low IOPs introduced the highest deviation from the GAT.

Performance of the M-TONX pachymetry was investigated using the Pentacam topography as the reference technology, and a very strong agreement was observed (>0.8). The difference between Pearson and Spearman correlation was not as high as the one for IOP, exhibiting further tendency to the Gaussian distribution for CCTs. A stable performance was observed over the CCT classes for different thickness ranges. The intraclass correlation (ICC) was calculated using Fisher formula, showing a very strong agreement for the overall IOP and CCT with ICC_IOP_ = 0.93 and ICC_CCT_ = 0.81, respectively.

Studies showed that the performance of the rebound tonometer, for example, iCare products, is noticeably influenced by the corneal thickness.^14^^,^^15^ In this study, we investigated the effect of the corneal thickness on the M-TONX tonometer for the 3 classes of the corneal thickness: class 1 (CCT < = 475 µm), class 2 (475 < CCT < 575 µm), and class 3 (CCT > 575 µm). This classification was performed based on the CCT values measured by the Pentacam. The IOP values, measured by M-TONX and GAT, were independently categorized into the three groups of IOP. The intraclass correlation among the groups was separately calculated for the three classes of CCT using the Cohen_Kappa formula. Table 3 lists the intraclass correlation of the M-TONX and the GAT along with the Pearson and Spearman correlation, and the reliability values. A substantial agreement was found for all the 3 classes of CCT with Cohen_Kappa value of larger than 0.6. The other metrics implicated on a stable performance of the M-TONX tonometry for a broad range of the CCT values. To obtain a quantitative understanding about the deviation of the M-TONX measurement from the reference devices, the GAT (for IOP) and the Pentacam (for CCT), the measurement deviations, namely *E_IOP_* and *E_CCT_*, were separately calculated and the corresponding statistics were estimated. Table 4 demonstrates the results.

(3) $$\begin{array}{c} E_{IOP}=IOP_{MTONX}-IOP_{Goldmann}\;\; \end{array}$$

(4) $$\begin{array}{c} E_{CCT}=CCT_{MTONX}-CCT_{Pentacam}\; \end{array}$$

**Table 3.** The Intraclass Correlation of IOP Calculated Using Cohen_Kappa (ck) Measure, for the 3 Classes of CCT Defined as: Class 1 (CCT < = 475 µm), Class 2 (475 < CCT < 575 µm), and Class 3 (CCT > = 575 µm)

		IOP, mm Hg			
		ck	r	Rs	rl
CCT, µm	Class 1	0.65	0.93	0.81	0.87
	Class 2	0.65	0.93	0.82	0.87
	Class 3	0.65	0.93	0.82	0.86

The agreement between the M-TONX and the Goldman measurements was calculated using the Pearson correlation (r), the Spearman correlation (rs), and the Reliability (rl).

**Table 4.** The Descriptive Statistics of the Measurement Deviations From the Standard Devices for the Full Span and the Stratified Ranges

	Overall	IOP < = 16	16 < IOP < 23	IOP > = 23	CCT < = 475	475 < CCT < 575	CCT > = 575
*E_IOP_*, mm Hg							
CI	0.7 to 1.2	1.0 to 1.5	−1.1 to −0.4	0.7 to 2.3	−0.4 to −1.7	0.8 to 1.6	0.8 to 2.3
IQR	3.1	3.0	3.0	2.9	3.9	3.0	3.3
Max	7.3	7.3	5.0	5.0	4.0	7.3	5.8
Mean	1.0	1.2	−0.3	1.5	0.7	1.2	1.5
Median	1.0	1.0	0.0	2.0	0.9	1.0	1.0
Min	−7.0	−4.5	−7.0	−5.0	−2.6	−0.6	−4.5
*P* value	0.0	0.01	0.10	0.01	0.13	0.17	0.13
Normality	N-N	N-N	N	N-N	N	N	N
S.S.	6	5	8	6	5	6	6
STD	2.3	2.1	2.7	2.5	2.3	2.3	2.3
*E_CCT_*, µm							
CI	−12.7 to −5.4	−13.1 to −3.7	−23.0 to −7.2	−7.6 to −17.4	3.2 to 26.6	−12.0 to −4.0	−32.3 to −16.9
IQR	33.0	37.0	18.5	31.0	46.0	29.3	34.5
Max	68.0	68.0	19.0	54.0	68.0	54.0	34.0
Mean	−9.0	−8.4	−15.1	14.9	14.9	−8.0	−24.6
Median	−9.0	−9.5	−11.5	13	15.0	−9.0	−27.5
Min	−76.0	−67.0	−70.0	−43.0	−33.0	−67.0	−76.0
*P* value	0.54	0.21	0.39	0.34	0.39	0.21	0.83
Normality	N	N	N	N	N	N	N
S.S.	5	5	4	5	6	3	5
STD	27.8	29.2	21.2	26.0	28.3	25.1	26.1

Upper row measurements by the GAT (IOP) and the Pentacam (CCT). In the statistics, the 95% confidence intervals, the interquartile range, and the standard deviation were denoted by CI, IQR, and STD, respectively. Normality of the variables’ distribution was tested using the Shapiro-Wilk method, and the *P* value of the hypothesis (accepting normal distribution) was independently listed in the table and denoted by *P* value. The statistical inference on normality of the distribution is indicated by N and N-N for the Normal and None-Normal distribution, using the significance level of 5%. The Sample Size (S.S.) was calculated based on the error margins of ±1 mm Hg and 25 µm, for the IOP and CCT, respectively.

**Figure 4.**
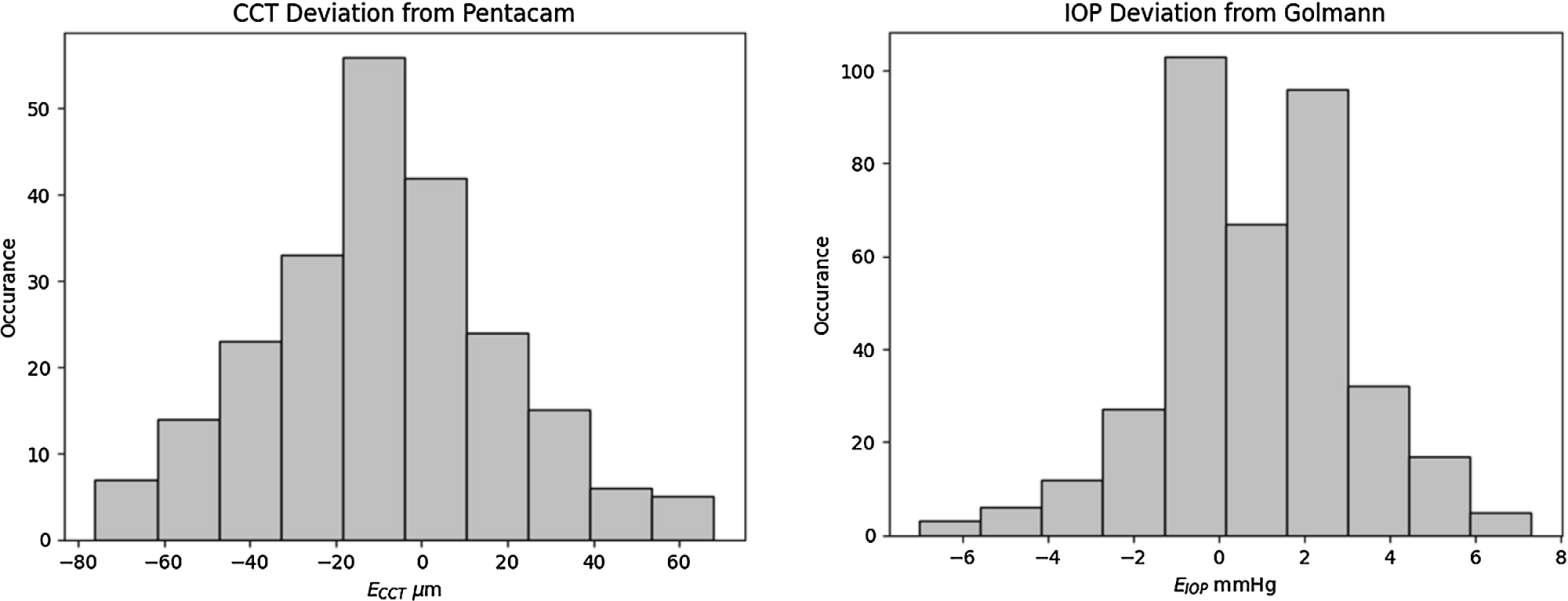
Histograms of the M-TONX measurement deviations from the standard devices; the Goldmann applanation tonometer (*left*) and the Pentacam topography (*right*).

The histograms of *E_IOP_* and *E_CCT_* are plotted in Figure 4. Table 4 demonstrates the results. The confidence interval (95% confidence level) of *E_IOP_* and *E_CCT_*, was estimated to be 0.7 to 1.2 mm Hg and −12.7 to −5.4 µm, for IOP and CCT, respectively, exhibiting an excellent measurement accuracy. The confidence intervals of *E_IOP_* and *E_CCT_* were estimated for all the three groups and the three classes. The stratified confidence intervals are listed in Table 4. The expected value of the overall IOP deviation is 1.0 mm Hg, whereas this value was minimal with *E_IOP_* = −0.3 mm Hg for the medium IOPs (16 < IOP < 23). The expected value of the deviation reached its maximal value for the high IOPs (IOP ≥ 23 mm Hg), and for the thick CCTs with the average *E_IOP_* = 1.5 mm Hg. The difference between the median and mean values was trivial for all the groups of IOP, implying on an acceptable number of the outliers. This was confirmed by the low difference between the standard deviation and the interquartile range for all the groups. Although normality was rejected for the overall IOP, and the groups with low, and with high IOPs (the *P* value of the medium IOP was 0.1), the standard deviation could still be used to estimate the confidence interval. The calculated sample sizes for introducing a margin of 2 mm Hg of error was relatively small. This is an indication of a good size of the study population. Moreover, the average value of the IOP deviations remained relatively stable over a broad range of IOP, and for all the three CCT classes. The CCT showed an average value of −9.0 µm deviation from the Pentacam, for the complete span of the IOP, whereas the high IOP group delivered the lowest deviation from the Pentacam.

The thick CCT group (CCT > 574 µm) exhibited the maximal deviation from the Pentacam with the average value of −24.6 µm. The thickness showed a tendency toward underestimation of CCT for a broad span of IOP and CCT. As like in the IOP cases, the median and the mean values introduced slight difference, and even more, the normality test was not rejected as the *P* values were typically high (see Table 4). Therefore, the confidence interval as well as the standard deviations were statistically significant. A small sample size needed for 25 µm of error margin (5% error) was small for all the CCT classes. This is an indication of incorporating a good number of the subjects in the study in terms of the CCT accuracy.

**Figure 5.**
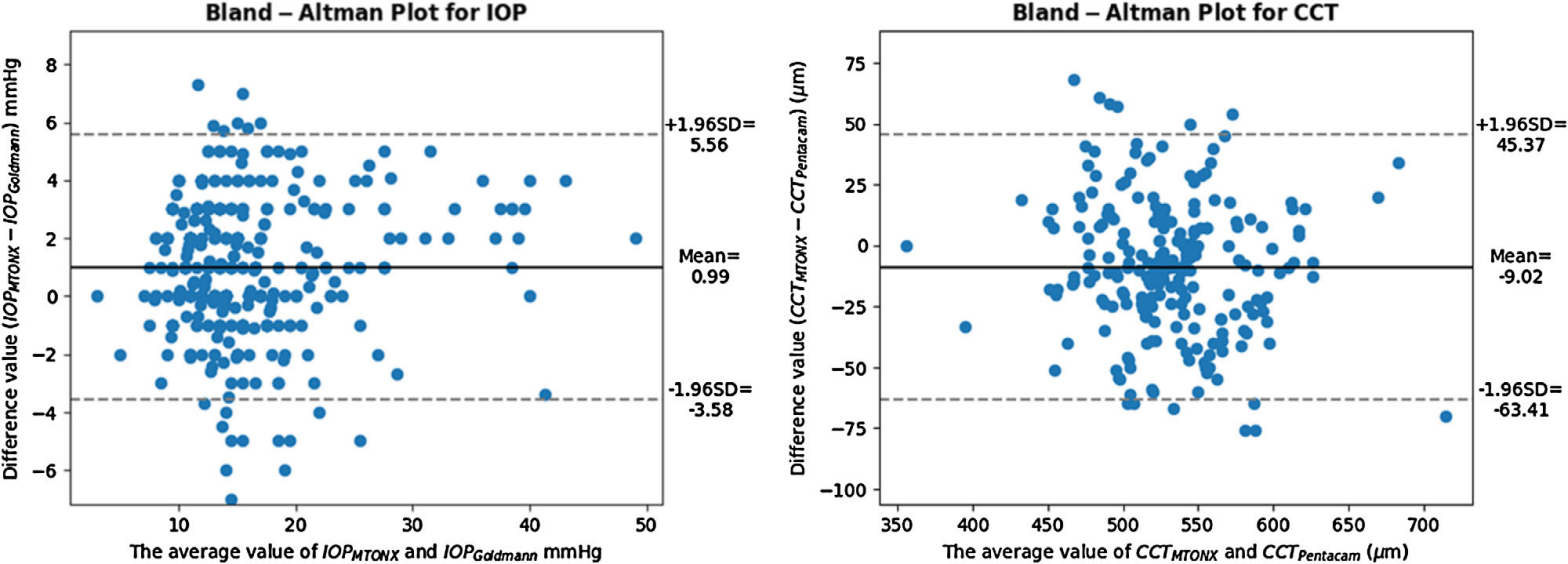
Bland-Altman plot for the IOP (*left*) and CCT (*right*), obtained for the M-TONX using the Goldman applanation tonometer and the Pentacam as the reference device for the IOP and CCT, respectively.

Figure 5 illustrates the Bland-Altman of IOP and CCT, separately obtained by the M-TONX tonometer and pachymeter, using the Goldmann and the Pentacam devices as the references.

The Bland-Altman plot, of IOP and CCT, showed a very strong agreement between the M-TONX and the GAT for the patients with high value, and for those with low value of IOP, whereas the mid-range IOPs comprised more outliers in terms of the deviation from the standard device. The performance for CCT showed limited deviation with lesser outliers. Table 5 represents the percentage of the IOP values meeting the deviation margin of 2, 4, and 5 mm Hg, along with the ones for CCT with the deviation margins of 25, 40, and 50 µm.

**Table 5.** Deviation of the IOP Measurements From the Goldmann Applanation, E_IOP_ (Equation 3), in Terms of the Percentage of the Measurements Fitting in the Defined Error Margins of ±2, ±4, and ±5 mm Hg

Deviation	Overall	Group 1	Group 2	Group 3
|*E_IOP_*| ≤ ±2 mm Hg	63.3%	66.2%	61.7%	47.6%
|*E_IOP_*| ≤ ±4 mm Hg	91.0%	93.2%	83.3%	88.1%
|*E_IOP_*| ≤ ±5 mm Hg	97.3%	97.4%	95.0%	100%

The percentages are represented for the full range (overall), group 1 (IOP < = 16 mm Hg), group 2 (16 < IOP < 23 mm Hg), and group 3 (IOP > = 23 mm Hg), respectively.

Table 5 represents the percentage of the data fits in the defined deviation margins of ±2, ±4, and ±5 mm Hg. The error margins fulfill the directive of ISO 8612. According to this standard, the margin of ±5 mm Hg covers the entire data of the group 4. This margin incorporated more than 95% of the data for all the groups. The error margin of ±2 and ±4 mm Hg comprised more than 63.3% and 91.0% of the data, respectively. The low IOP group exhibited the minimal deviation. Table 6 represents the percentage of the data admitted by the different deviation margins of CCT measurement. In about 17% of the measurements, the M-TONX showed a deviation of more than 40 µm from the Pentacam. In most of the cases, the patients did not show an appropriate cooperation with the research operators. Among the three classes, the optimal performance was seen for the medium CCT range.

**Table 6.** Deviation of the CCT Measurements From the Pentacam Topography, E_IOP_ (Equation 4), in Terms of the Percentage of the Measurements Fitting in the Defined Error Margins of ±25, ±40, and ±50 µm

Deviation	Overall	Class 1	Class 2	Class 3
|*E_CCT_*| ≤ ±25 µm	64.0%	64.0%	69.7%	45.8%
|*E_CCT_*| ≤ ±40 µm	82.7%	80.0%	86.2%	72.9%
|*E_CCT_*| ≤ ±50 µm	90.2%	84.0%	92.8%	85.4%

The percentages are represented for the full range (overall), class 1 (CCT < = 25 µm), class 2 (475 < CCT < 575 µm), and class 3 (CCT > = 474 µm), respectively.

## Discussion

This paper presents the results of an investigation on a newly introduced device for ophthalmic measurements; the M-TONX. This user-friendly and patient-friendly device combines bound and rebound technology, serving as both a tonometer and a pachymeter. The dedicated probe of the device gently depresses the center of cornea with a low pressure, below the threshold to initiate any adverse situation. This was confirmed by the practical investigations, where some of the subjects did not even sense the probe.^19^

In this investigation, we aimed to evaluate the quality of the M-TONX by applying appropriate standards and conducting a clinical study using well-established medical devices. The investigation on the M-TONX was conducted in accordance with the directives of ISO 8612, where all the corresponding requirements were met. A very strong correlation was observed between the M-TONX measurements and those from the standard device for both IOP and CCT. The device provided an expectedly stable performance over different ranges of IOP and CCT. The overall deviation of the M-TONX from the GAT was expected to be 1 mm Hg toward overestimation, which was favorable to the ophthalmic investigations according to the clinical praxis. The overestimation was further observed in the eyes with high IOP (group 3), and relatively stable over the CCT classes (classes 1 and 2). This is a desirable feature when it comes with the glaucoma patients with thin cornea. It is worth mentioning that the M-TONX measures IOP relying on the temporal interval between the time when the probe begins to touch the corneal surface and the time when the probe movement is completely damped by the cornea. Therefore, the CCT plays a role in the speed decline of the moving probe, which turns out to affect the speed-time profile of the probe movement. The overall deviation of the M-TONX from the Pentacam was expected to be 14 µm in measuring CCT, but toward underestimation.

The IOP was slightly underestimated for the medium IOPs (group 2). In contrast, the CCT was underestimated across all ranges of IOP and CCT, except for two subgroups: those with low CCT values (CCT < 475 µm) and those with high IOP values (IOP > = 23 mm Hg). The maximal deviation of CCT from the Pentacam, was seen in class 3 (thick CCTs) and group 2 (medium IOPs; see Fig. 4). The high IOPs also introduced a higher deviation from the GAT than the low IOPs toward overestimation (see Fig. 4). Nevertheless, the CCT value did not noticeably affect the IOP deviation (see Fig. 4).

In comparison to the other rebound-based devices, the M-TONX exhibited superior performance. This is clearly achievable considering the advanced technology deployed in the device. For example, the M-TONX is uniquely equipped with an optical alignment technology, which precisely directs the light beam at the probe tip. This helps the users to find the corneal center in an easy and convenient manner. Moreover, the M-TONX uses advanced machined learning methods in conjunction with an improved measurement technology in contrast with the other rebound-based devices, such as the iCare Pro.

The correlation coefficient of the iCare Pro with the GAT was reported to be 0.673 by Tamelik et al.^14^ The study was conducted on large group of patients with a wide range of IOP and CCT. They concluded that the iCare Pro tends to overestimate IOP for the low GAT-measured IOPs (IOP < 8 mm Hg), in contrary with the high IOPs (GAT-measured), which were underestimated by the iCare Pro.^14^ The study reported an agreement of ±5 mm Hg in 79.6% of the cases with IOPs between 22 and 29 mm Hg, and in 36.4% of the cases with IOP > 30 mm Hg. The best agreement was found in the IOPs within 9 to 22 mm Hg (60.7% of the measurements were within the range of ±2 mm Hg). Comparing the outcomes of the study by Tamelik et al.^14^ to the findings of this study, that is, Table 5, the outperformance of the M-TONX over the iCare is considerable.

**Figure 6.**
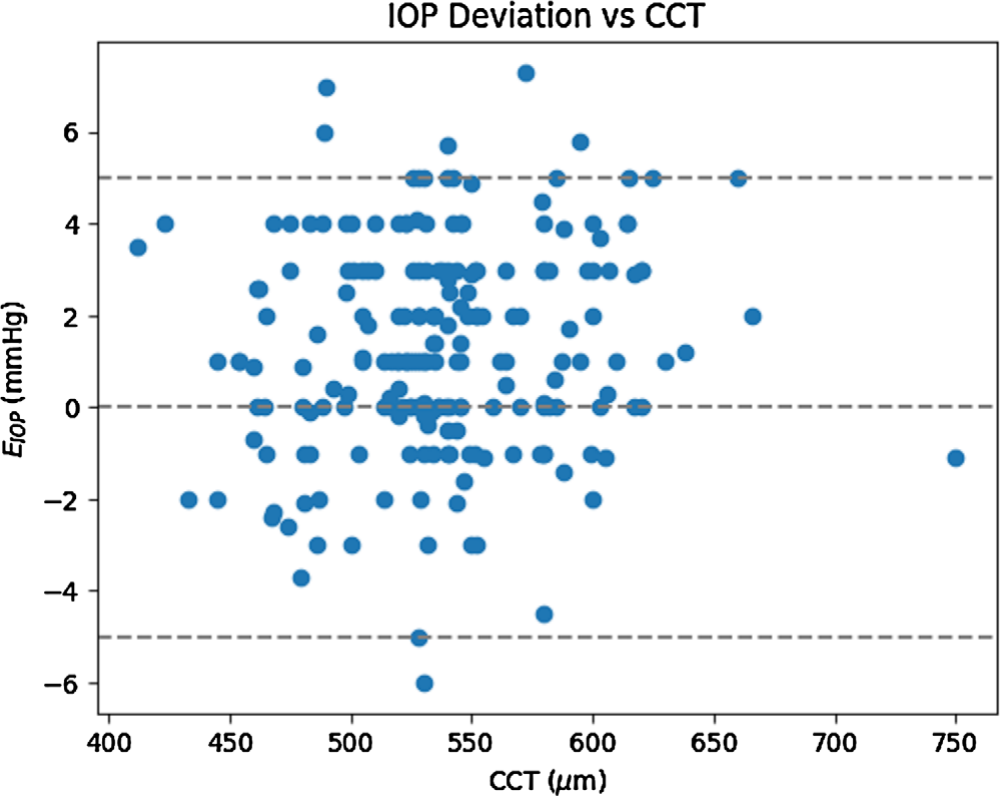
Distribution of the M-TONX deviation from Goldmann applanation tonometer with respect to the CCT measured by the Pentacam.

Kim et al. also found that the IOP deviation of the iCare from the GAT proportionally decreases with IOP.^20^ In contrary, Chui et al. found that the iCare overestimates high IOPs and underestimates low IOPs using the GAT as the reference device.^21^ To some extent, this is in concordance with our findings with the M-TONX. However, the effect of CCT cannot be evidently undermined. Figure 6 illustrates the scatter plot of *E_IOP_* and CCT (measured by the Pentacam).

As seen in Figure 6, the M-TONX deviation exhibits a uniform distribution over a broad span of CCT without a noticeable bias for the thick corneas, unlike the iCare tonometer in which corneal thickness considerably affects the IOP measurement. Discrepancy between the iCare and the GAT in IOPs ≥ 21 mm Hg was also reported by Pakrou et al.^22^ This point was addressed in a different study by Gao et al. performed on 336 patients.^23^ The patients of that study were divided into 3 groups according to their GAT-measured IOPs: group A = 7 to 15 mm Hg, group B = 16 to 22 mm Hg, and group C = 23 to 50 mm Hg. The 95% confidence interval of the deviation from the GAT measurements was estimated to be −5.80 to 6.24 mm Hg.^23^ The iCare measurements were significantly lower (−1.66 ± 3.87 mm Hg) than the GAT ones, for IOP ≥ 23 mm Hg. This is an alarming finding pinpointing likelihood of misdiagnosing glaucoma patients. In group A and B cases, this difference was smaller than in the group C (−0.12 ± 2.77 and 0.04 ± 2.86 mm Hg, respectively). Both the iCare (*r* = 0.390, *P* = 0.001) and the GAT (*r* = 0.191, *P* = 0.001) showed positive correlation with CCT.^23^ However, the correlation was not significant in the patients with high IOP, and the iCare readings were further influenced by CCT than the readings from the GAT.^23^ Accuracy of the iCare ic100 was explored by Subramaniam et al. over a large group of 1000 eyes using the GAT as the reference device.^24^ They reported a good correlation between the iCare ic100 and the GAT measurements, with an overall intra class correlations of 0.73, but the iCare ic100 consistently undermeasured the IOP compared to the GAT, with a mean difference of –4.2 mm Hg (standard deviation [SD] = ±4.3). Nevertheless, the intraclass correlation is considerably less than the one calculated for the M-TONX with *ICC__IOP_* = 0.93 (see the Results section). The mean difference was estimated to range from −1 to −2 mm Hg for IOPs ≤ 12 mm Hg, and from −6 to −7 mm Hg for IOPs ≥ 22 mm Hg.^25^ This range never exceeded 2 mm Hg for the M-TONX.

In our study, the M-TONX averagely overestimated the GAT by 1 mm Hg and this overestimation was more in the higher IOPs. In contrast to the findings of the previous study, and in concordance with our findings, Wong et al. compared the performance of the iCare ic100 to a GAT in 74 patients with glaucoma and glaucoma-suspect patients and the mean difference between the iCare ic100 and the GAT was 0.44 mm Hg with 95% of confidence interval of –8.18 to 9.06 mm Hg.^26^ Underestimation of the iCare ic100 was also confirmed by Nakukura et al. in a comparative study on the ic100 and the GAT.^27^ The mean deviation from the GAT was estimated to be −4.24 mm Hg. They also found a negative correlation of IOP values between the rebound tonometer (iCare ic100 and iCare TA01i) and age, which was not observed in the GAT measurements. Moreover, low CCTs and small corneal curvature introduced further underestimation into the iCare measurements even for the patients with glaucoma.^27^ The underestimation was observed for both the iCare ic200 and the iCare Pro, especially for the low IOPs.^28^ This was in contradiction to the results obtained by Badakere et al. in which the iCare 200 overestimated the IOPs compared to the GAT.^29^ The overestimation proportionally increased by the baseline IOP.^29^ Several factors may explain the different results, including the biomechanical properties of the cornea, which can be affected by use of IOP lowering drops commonly used by patients with glaucoma, as well as the design and size of the probe in various rebound tonometers. In contrast, the M-TONX offered relatively stable accuracy for various CCT values (see Fig. 6).

Our study had some limitations. First, we did not include patients with corneal pathology, such as edema and opacity, or post corneal graft surgeries. As such, the findings of our study may not be applicable to these group of the patients. Second, we did not conduct a comparative study on the M-TONX and either the iCare 100 or the iCare 200. The comparison was performed using the scientific publications on the iCare ic100 and the iCare ic200, for the similar patient groups. Nevertheless, the present study incorporated solid aspects, such as the good number of the study subjects and the class analysis of patients with different CCTs and different IOPs (see Table 2; Table 4). As can be seen in Table 4, the sample sizes needed to secure reproducibility of the results (95% confidence interval), are reasonably small with a narrow margin of error for all the IOP groups and the CCT classes.

## Conclusions

The M-TONX is the first rebound tonometer pachymeter device with the ability to measure IOP and CCT simultaneously. It serves as an accurate measuring device, which benefits from the advanced technology used for the data acquisition and processing. Our investigation on the M-TONX showed a stable performance with a very strong correlation between the measurements that resulted from the M-TONX and the ones from the standard devices. The study met all the requirements of ISO 8612 for ophthalmic investigations.
